# Cost-effectiveness of Direct Transfer to Angiography Suite of Patients With Suspected Large Vessel Occlusion

**DOI:** 10.1212/WNL.0000000000207583

**Published:** 2023-09-05

**Authors:** Chi P. Nguyen, Maarten M.H. Lahr, Durk-Jouke van der Zee, Henk van Voorst, Marc Ribo, Yvo B.W.M. Roos, Ido van den Wijngaard, Erik Buskens, Maarten Uyttenboogaart

**Affiliations:** From the Department of Operations (C.P.N., D.-J.v.d.Z., E.B.), Faculty of Economics and Business, University of Groningen; Health Technology Assessment (C.P.N., M.M.H.L., D.-J.v.d.Z., E.B.), Department of Epidemiology, and Departments of Neurology (M.U.), and Radiology, Medical Imaging Center (M.U.), University of Groningen, University Medical Center Groningen, the Netherlands; Department of Pharmaceutical Administration and Economics (C.P.N.), Hanoi University of Pharmacy, Vietnam; Departments of Radiology and Nuclear Medicine (H.v.V.), Biomedical Engineering and Physics (H.v.V.), and Neurology (Y.B.W.M.R.), Amsterdam University Medical Center, the Netherlands; Unitat d'Ictus (M.R.), Servei de Neurologia, Hospital Universitari Vall d'Hebron, Spain; Department of Neurology (I.v.d.W.), Haaglanden Medical Center; and Department of Neurology (I.v.d.W.), Leiden University Medical Center.

## Abstract

**Background and Objectives:**

Patients with acute ischemic stroke due to large vessel occlusion (LVO) deemed eligible for endovascular thrombectomy (EVT) are transferred from the emergency room to the angiography suite to undergo the procedure. Recently, the strategy of direct transfer of patients with suspected LVO to the angiography suite (DTAS) has been shown to improve functional outcomes. This study aims to evaluate the cost-effectiveness of the DTAS strategy vs initial transfer of patients with suspected LVO (Rapid Arterial Occlusion Evaluation score >4 and NIH Stroke Scale >10) to the emergency room (ITER).

**Methods:**

A decision-analytic Markov model was developed to estimate the cost-effectiveness of the DTAS strategy vs the ITER strategy from a Dutch health care perspective with a 10-year time horizon. The primary outcome was the incremental cost-effectiveness ratio (ICER) using Dutch thresholds of $59,135 (€50,000) and $94,616 (€80,000) per quality-adjusted life year (QALY). Uncertainty of input parameters was assessed using 1-way sensitivity analysis, scenario analysis, and probabilistic sensitivity analysis.

**Results:**

The DTAS strategy yielded 0.65 additional QALYs at an additional $16,089, resulting in an ICER of $24,925/QALY compared with the ITER strategy. The ICER varied from $27,169 to $38,325/QALY across different scenarios. The probabilistic sensitivity analysis showed that the DTAS strategy had a 91.8% and 97.0% likelihood of being cost-effective at a decision threshold of $59,135/QALY and $94,616/QALY, respectively.

**Discussion:**

The cost-effectiveness of the DTAS strategy over ITER is robust for patients with suspected LVO. Together with recently published clinical results, this means that implementation of the DTAS strategy may be considered to improve the workflow and outcome of EVT.

## Introduction

The mean prevalence of large vessel occlusion (LVO) in acute ischemic stroke is approximately 30% (7%–61%), and the presence of LVO in acute ischemic stroke is associated with a higher risk of death compared with non-LVO.^1^ Endovascular thrombectomy (EVT) has become the standard treatment for acute ischemic stroke due to anterior circulation LVO. EVT was cost-effective compared with medical treatment in various countries,^2^ but the effect remains highly time-dependent.^3^ Faster in-hospital processes of acute stroke care, such as emergency department door-to-reperfusion time, are associated with better 90-day functional outcomes for patients with LVO treated with EVT.^3^ In fact, 10 minutes of faster EVT resulted in 13.5 disability-free life days saved in Dutch patients^4^ and 39 disability-free life days in the US patients.^5^ Therefore, optimal EVT treatment necessitates expediting the diagnostic process of acute stroke.

In most Western countries, LVOs are usually diagnosed with CT angiography (CTA) performed in the emergency department. When deemed eligible, patients will then be transferred to the angiography suite to undergo EVT. However, a flat-panel CT in the angiography suite also can rule out intracranial hemorrhage or large established ischemic lesions, which contraindicate EVT. After flat-panel CT, LVO can be confirmed by digital subtraction angiography.^6^ Recently, several studies have shown that direct transfer to the angiography suite (DTAS) of patients with suspected LVO significantly decreased hospital workflow time and improved functional outcomes at 90 days for patients with LVO.^6,7^ DTAS opens a potential strategy of workflow improvement for rapid delivery of EVT bypassing CTA in the emergency department. However, introducing DTAS could result in higher costs and unnecessary occupation of the angiography suite in non-LVO patients and increase the workload for the angiography team. A recent economic evaluation^8^ concluded that DTAS was cost-effective in patients with suspected LVO within 6 hours from stroke onset in Spain. Therefore, the aim of this study was to evaluate the cost-effectiveness of DTAS compared with initial transfer to the emergency room (ITER) in patients with suspected LVO from the Dutch health care perspective.

## Methods

### Population, Strategies, and Setting

The Evaluation of Direct Transfer to Angiography Suite vs. Computed Tomography Suite in Endovascular Treatment: Randomized Clinical Trial (ANGIOCAT) trial^6^ was an open, randomized controlled trial with blinded end point assessment conducted in Spain. The study evaluated the functional outcome at 90 days after EVT for patients with LVO after the DTAS or ITER workflow. The trial included 174 patients with suspected LVO^6^ within 6 hours of symptom onset who had a Rapid Arterial Occlusion Evaluation (RACE) score >4 on scene or during transport and NIH Stroke Scale (NIHSS) >10 at arrival as scored by neurologists. LVO was confirmed in 147 patients (74 in DTAS and 73 in ITER).^6^ Based on the characteristics of patients in the ANGIOCAT trial,^6^ we generated a hypothetical cohort of 10,000 patients with suspected LVO with a mean age of 74 years. This was necessary as the DTAS strategy is currently no routine clinical practice in the Netherlands. In the DTAS strategy, patients with suspected LVO (RACE >4 and NIHSS >10) would be directly transferred to the angiography suite for diagnostic evaluation, potentially followed with IV thrombolysis (IVT) and EVT. A flat-panel CT was performed to rule out intracranial hemorrhage or a large established infarct. Subsequently, IVT could be administered. Patients would undergo EVT if angiography confirmed LVO. In the ITER strategy, patients with suspected LVO were first transferred to the emergency room to confirm LVO by CT or CTA. If necessary, the treating physician could decide for the use of CT perfusion. When indicated, IVT would be administered directly after CT or CTA. Next, patients would be transferred to the angiography suite to undergo EVT when deemed eligible. Both strategies were applied to (hypothetical) patients directly admitted to comprehensive stroke centers in the Netherlands.

### Model Structure and Assumption

**Figure 1:**
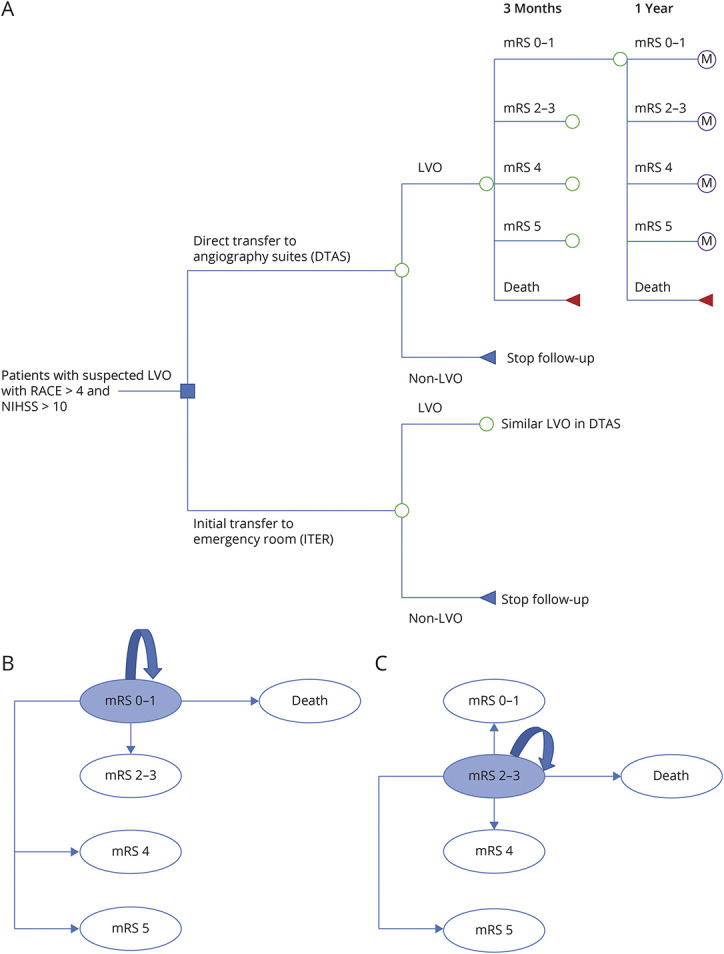
Cost-effectiveness Model Structure (A) Decision tree mode. (B) 1-year cycle Markov model for patients with mRS 0–1. (C) 1-year cycle Markov model for patients with mRS 2–3. LVO = large vessel occlusion; M = Markov model; mRS = modified Rankin scale; RACE = Rapid Arterial Occlusion Evaluation.

We developed a 1-year decision tree model and a 9-year Markov model to estimate the cost-effectiveness of the DTAS strategy vs the ITER strategy from a Dutch health care payer perspective (Figure 1). A 10-year model was chosen because the average life expectancy for a 75-year person was 12 years in the Netherlands.^9^ We assumed that workflow efficiencies and adverse events between the 2 strategies would be similar for non-LVO patients,^6^ but diagnosis costs in non-LVO patients were taken into account in the model.

Functional outcome is assessed 90 days after stroke for the modified Rankin Scale (mRS) score, with higher scores indicating more severe disability. The DTAS and ITER strategies resulted in different mRS scores at 90 days after stroke in the ANGIOCAT trial.^6^ Recently, clinical trials reported excellent function (combining mRS 0 and mRS 1) as the primary outcome in stroke patients.^10^ In addition, mRS 2 and mRS 3 had similar health utility and costs.^4,11^ Hence, we included 5 states in our model: mRS 0–1, mRS 2–3, mRS 4, mRS 5, and death (mRS 6). We assumed that the mRS at 90 days might significantly improve or deteriorate in the first year after stroke but would remain stable thereafter. Therefore, patients' mRS at 1 year was used as an entry state in the Markov model to estimate long-term outcomes in the subsequent 9 years. During each 1-year cycle, patients could remain in their mRS or make a transition to another mRS or “death due to all causes” (Figure 1, B and C). In the Markov model, long-term costs and effectiveness were attributed to mRS scores, and we assumed that other risk factors would be similar between groups.

### Input Parameters

#### Epidemiology and Clinical Parameters

We identified 7 relevant studies comparing DTAS and ITER based on the recent systematic review.^12^ However, 1 trial^13^ did not report mRS score at 90 days, 1 case-control study^14^ reported mRS 0–2 only, and 4 studies^15-18^ had an observational design and were conducted in patients admitted first to primary stroke centers and then transferred to EVT-capable centers. Therefore, the ANGIOCAT trial^6^ was the only included study to assess short-term outcomes for patients in the DTAS and ITER groups (Table 1). The transition matrix of changing mRS from 90 days to 1 year and from 1 year onward was identified by applying data from the extended follow-up of the MR CLEAN trial^19^ (eTables 1–3, links.lww.com/WNL/C984). Quality of life, expressed in utility, was derived from a previous publication for the Dutch stroke population.^4^

**Table 1:**
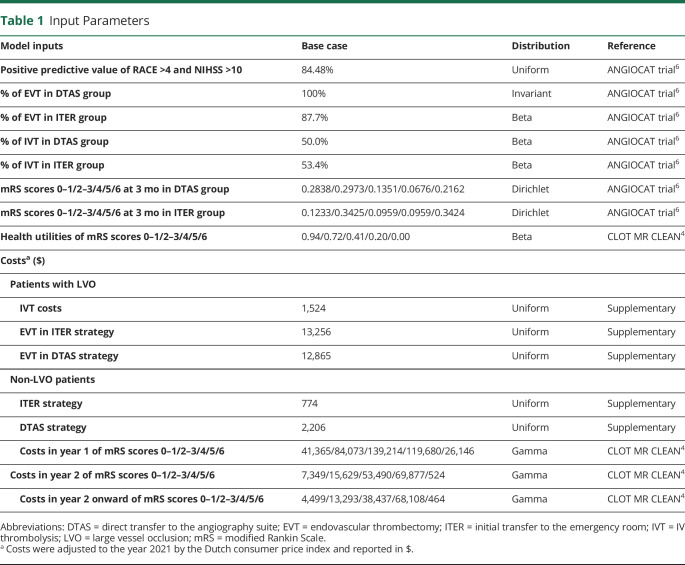
Input Parameters

The mortality rate in the Markov model was estimated by combining results of the clinical trial^19^ and mortality data of the Dutch life tables for different age groups.^20^ The age-based risk for mortality for the Dutch general population was derived from Dutch survival statistics (Central Bureau of Statistics).^20^ We assumed that the mortality rate over the 10-year period after stroke depended on the mRS and age (eTable 3, links.lww.com/WNL/C984).

#### Cost Parameters

Direct medical costs were included in our models based on a Dutch health care perspective. Health care costs in LVO and non-LVO patients were derived from data repositories and expert opinion from the University Medical Center Groningen, published data, and the Dutch reference costs (Table 1 and eTable 4, links.lww.com/WNL/C984). All costs were converted to the reference year 2021 in Euros (€) by using the Dutch consumer price index^21^ and reported in USD ($) (€1 = $1.1827 in 2021).^22^

### Outcomes

The primary outcome was the incremental cost-effectiveness ratio (ICER) calculated as the difference in the total costs between 2 strategies divided by the difference in the quality-adjusted life years (QALYs). The DTAS strategy was considered cost-effective if the ICER fell below the decision threshold of €80,000/QALY ($94,616).^23^ In addition, we applied the decision threshold of €50,000/QALY (per capita Gross Domestic Product per QALY) ($59,135) to consider the DTAS highly cost-effective in line with the World Health Organization recommendations.^24^ In accordance with the Dutch guideline for economic evaluations in health care, we applied an annual discount rate of 4% and 1.5% for costs and QALYs, respectively, to take into account the effect of time preference on costs and effectiveness.^25^ All analyses and modeling were performed in Treeage Pro 2022 R1.2. The study was reported according to the Consolidated Health Economic Evaluation Reporting Standards statement.^26^

### Scenario Analyses

Patients with suspected LVO with RACE score >4 and NIHSS >10 were included in the base case analysis as they were inclusion criteria in the ANGIOCAT trial.^6^ To explore the effect of inclusion criteria (i.e., without NIHSS >10 on admission), we applied the positive predictive value of RACE score >4 (0.42) from the PRESTO trial in scenario 1.^27^ Currently, the prehospital RACE scale is not routinely used in the Netherlands (except for some regions).^28^ Therefore, we also analyzed the cost-effectiveness of the DTAS vs ITER strategies in suspected stroke patients by EMS paramedics with positive face-arm-speech-time (FAST) test^27^ (without the results of the RACE scale) to accurately reflect the current situation in the Netherlands (scenario 2). An incidence of 12% LVO in the cohort of suspected stroke patients was used in scenario 2 as a conservative assumption.^27^ In addition, a 4-year Markov model was applied for scenario 3 to further explore the effect of follow-up time on the strategies.

### Sensitivity Analyses

We employed 1-way sensitivity analyses to assess the effect of uncertainty of the input parameters on the ICER. Input parameters with their range are summarized in the eTable 5 (links.lww.com/WNL/C984). Parameters for which sensitivity analysis indicated a high effect on the ICER were presented in a Tornado diagram.

A probabilistic sensitivity analysis was performed using Monte Carlo simulation with 10,000 iterations by drawing random values from input parameters' distribution to evaluate uncertainty (Table 1 and eTable 6, links.lww.com/WNL/C984). The probabilities of each strategy being cost-effective at varying decision thresholds were presented using cost-effectiveness acceptability curves.

### Model Validation

We applied the Assessment of the Validation Status of Health Economic decision models (AdViSHE) tool^29^ in the process of model validation. The AdViSHE tool includes 13 items divided into 5 parts: validation of the conceptual model, input data validation, validation of the computerized model, operational validation, and other validation techniques.

### Standard Protocol Approvals, Registrations, and Patient Consents

Ethics approval was not required as this work was based on a model-based analysis with published input parameters, and no individual patient data were used. This study was not registered.

### Data Availability

All relevant data are provided in the manuscript and its supplementary material.

## Results

### Base Case and Scenario Analyses

The DTAS strategy resulted in higher total costs ($16,089) but also yielded 0.65 more QALYs compared with the ITER strategy for each patient with suspected LVO. Therefore, the DTAS strategy was cost-effective with an ICER of $24,925 per QALY gained, that is, well below the decision threshold of $59,135 and $94,616. In the scenario analyses, the DTAS strategy remained a beneficial strategy also in a setting not using NIHSS >10 (an ICER of $27,169 per QALY) and without prehospital RACE (an ICER of $38,325 per QALY) in all suspected stroke patients. Furthermore, when a 4-year Markov model (scenario 3) was applied, the DTAS strategy remained the cost-effective strategy compared with the ITER strategy with an ICER of $34,050 per QALY (Table 2).

**Table 2:**
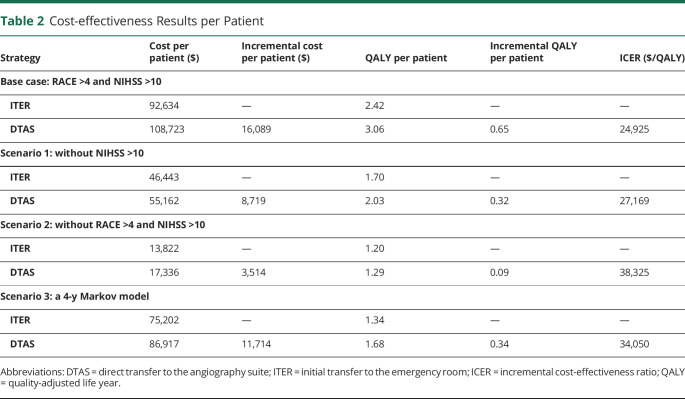
Cost-effectiveness Results per Patient

### Sensitivity Analyses

One-way sensitivity analyses showed that the mortality rate in the ITER group, the proportion of mRS 0–1 and mRS 4 in the DTAS group, and the costs of EVT had a high effect on the ICER (eFigure 1, links.lww.com/WNL/C984). The mortality rate in the ITER group was the most influential variable on the ICERs ranging from $18,407 to $27,958. Similarly, when the proportion of mRS 0–1 in the DTAS group was adjusted from a minimum value of 22.70% to a maximum value of 34.06%, ICERs ranged from $30,567 to $21,322, respectively. The ICERs in 1-way sensitivity analysis varied from $18,407 to $30,567 but remained far below the threshold of $94,616 (eTable 7).

**Figure 2:**
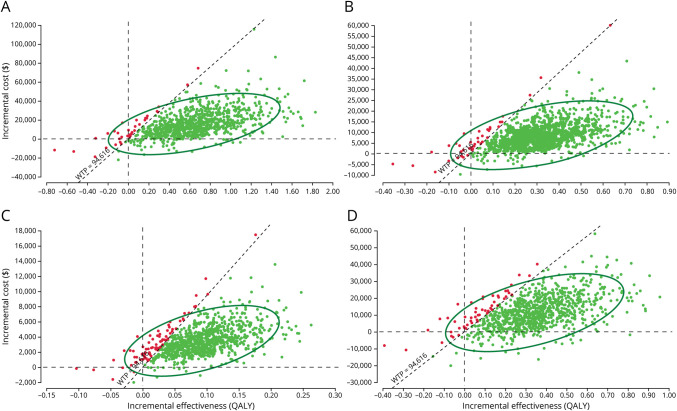
Probabilistic Sensitivity Analysis: Cost-effectiveness Plane (A) Base case. (B) Scenario 1. (C) Scenario 2. (D) Scenario 3. QALY = quality-adjusted life year.

**Figure 3:**
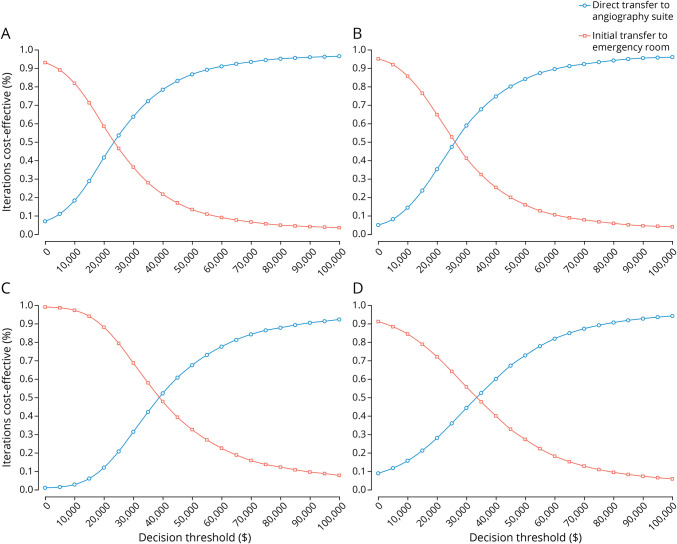
Cost-effectiveness Acceptability Curves at Different Decision Thresholds (A) Base case. (B) Scenario 1. (C) Scenario 2. (D) Scenario 3.

The scatter plots of probabilistic sensitivity analyses showed that under the decision threshold of $94,616, DTAS in base case, scenario 1, scenario 2, and scenario 3 had the corresponding 97.0%, 95.7%, 91.3%, and 93.4% probabilities of economic advantages,repectively (Figure 2). Figure 3 demonstrates a probability of being cost-effective for DTAS and ITER strategies based on different decision thresholds. In the base case, when the threshold ranged from $59,135 to $94,616, the DTAS strategy was cost-effective for 91.8%–97.0% of the simulations, respectively (Figure 3A). Probabilistic sensitivity analyses showed the robustness of cost-effectiveness of the DTAS strategy in scenarios (Figure 3, B–D). Model validation based on the AdViSHE tool is provided in the eAppendix 1 (links.lww.com/WNL/C984).

## Discussion

Our results indicate that the DTAS strategy is cost-effective over a period of 10 years compared with the ITER strategy, with an ICER of $24,925, for patients with suspected LVO with RACE >4 and NIHSS >10 within 6 hours of stroke onset. Although the DTAS strategy was more costly than the ITER strategy, the DTAS strategy yielded 0.65 more QALYs and better functional outcomes at 90 days after stroke. Our findings are in line with previously published studies showing that earlier EVT treatment results in more disability-free life.^4,5^ A recent study from Spain reported that the DTAS strategy was dominant (lower costs and more QALYs) compared with the ITER strategy,^8^ whereas the ICER of DTAS was $24,925 in the Netherlands. Small differences in incremental QALYs were reported in the previous study^8^ (0.82 QALYs in lifetime horizon) compared with our findings (0.65 QALYs in 10 years). However, the DTAS strategy appeared to save €51,505 per patient in Spain while DTAS would add costs ($16,089) in our study. Different long-term costs and the model structure may explain these differences. For example, the Spanish study included 7 health states (mRS 0, mRS 1, mRS 2, mRS 3, mRS 4, mRS 5, and mRS 6) without taking into account possible mRS improvement after 90 days. Our model contained 5 health states (mRS 0–1, mRS 2–3, mRS 4, mRS 5, and mRS 6) while allowing actually observed mRS improvement.

Improvement of clinical outcomes in the DTAS group is most likely explained by less time delays in the DTAS approach (door-to-puncture time of 18 minutes in the DTAS group vs 42 minutes in the ITER group).^6^ Another potential explanation for better outcomes observed in the DTAS group is a higher rate of EVT in the DTAS group compared with the ITER group in the ANGIOCAT trial (100% vs 87.7%). Recently, the pooled results of 8 studies in a systematic review also showed that the DTAS strategy reduced door-to-puncture (29-minute reduction) and door-to-reperfusion times (32-minute reduction) compared with the ITER strategy. In addition, the DTAS strategy tended to be associated with better functional outcomes at 90 days (odds ratio [OR] 1.38, 95% CI 0.97–1.95) without any difference in 90-day mortality between the 2 strategies (OR 0.74, 95% CI 0.48–1.15).^30^ Results of ongoing trials comparing the DTAS and ITER strategies will provide more evidence on the effect of DTAS on 90-day outcomes.^31,32^

One-way sensitivity analysis and probabilistic sensitivity analysis support the robustness of our results. Cost-effectiveness was sensitive to the mortality rate in the ITER, the proportion of mRS 0–1, mRS4 in the DTAS, and the costs of EVT. The DTAS strategy remained beneficial, with an ICER still below the threshold of $94,616 when variables increased or decreased by 20%. In addition, the DTAS strategy was cost-effective in the scenario in which the DTAS approach was applied to all suspected stroke patients with a positive FAST test, reflecting current practice in large parts of the Netherlands. However, introducing the DTAS strategy for all suspected stroke patients will markedly increase the workload for the angiography team and lead to the unnecessary occupation of the angiography suite by non-LVO patients, making it unfeasible for standard use in clinical practice. Furthermore, the DTAS strategy increases the risk of adverse events, such as groin hematoma. Therefore, the DTAS strategy may be considered for patients with a high likelihood of LVO that is identified through triage using acceptable accuracy prehospital stroke scales. That may make the DTAS strategy more feasible in clinical practice. RACE is one of the best performing prehospital stroke scales to detect patients with LVO in the Netherlands.^27,28^ In addition, the DTAS strategy should be implemented for patients with LVO potentially having the most benefit from the DTAS strategy, thus avoiding angiography suite overload. For example, Requena et al.^33^ indicated that the DTAS approach had the largest effect on patients with LVO admitted in the early time window, from 0 to 3 hours after symptom onset. As such, the DTAS approach is potentially introduced in the highly patients with suspected LVO (RACE >4 and NIHSS >10) in the early window. In addition, broad EVT criteria (i.e., low RACE score with LVO, late window) for applying the DTAS fall beyond the scope of our current analysis.

Our model integrates the mRS score at 3 months and 1 year after stroke onset to estimate long-term outcomes. The mRS 3-month poststroke is commonly measured as the end point of randomized controlled trials and used as a proxy for modeling long-term clinical and economic outcomes in previous cost-effectiveness studies.^34,35^ These studies assumed that patients would not improve on the mRS beyond 90-day poststroke. However, more recent studies suggest that intermediate-term outcomes fluctuate over time, showing that from 91 days up to 365 days, patients may improve, remain in the same state, or even deteriorate.^36,37^ As such, the overall effect of new strategies might be overestimated or underestimated. The incorporation of short-term outcomes (90 days after stroke) and middle-term outcomes (1 year after stroke) in our model provides a more robust extrapolation of long-term stroke consequences. This approach is supported by evidence from the extension of follow-up patients in the MR CLEAN trial.^19^

A systematic review on poststroke care reported that rehabilitation services were the main cost driver of stroke burden.^38^ Therefore, the cost-effectiveness results of DTAS in the Netherlands suggest that DTAS is potentially cost-effective in other settings with a high stroke burden. For example, the poststroke care costs were highest in the United States ($4,644 per patient month), followed by Denmark ($3,026), the Netherlands ($2,214), and Norway ($2,147).^38^ In addition, 10-minute earlier EVT provided better net monetary benefit in the United States ($10,593)^5^ compared with the Netherlands (€3,085).^4^ Other factors, such as the prevalence of LVO and sensitivity of prehospital triage, may contribute to the feasibility of introducing DTAS in clinical practice. The presence of LVO in the stroke population seems similar in the Netherlands (12%)^27^ and the United States (11%).^39^ The American Heart Association and American Stroke Association recommends stroke scales to predict LVO, such as Cincinnati Prehospital Stroke Scale, Los Angeles Motor Scale, RACE, and Facial palsy, Arm weakness, Speech changes, Time, Eye deviation, Denial/neglect scale. Given the comparable performance of these (0.57–0.67), each emergency medical service region should choose an easy-to-use tool and monitor adherence and accuracy of the prehospital scale.^40^ In addition, our model with published input parameters may be used in other settings to assess the cost-effectiveness of the DTAS strategy at different decision thresholds (i.e., $100,000 per QALY in the United States,^41^ €22,000–€33,000 per QALY in Denmark^42^). Future work should confirm the economic benefit of the DTAS strategy in other settings.

This study has some limitations. First, we used mRS at 3 months from the ANGIOCAT trial in Spain because of the lack of data from the Netherlands. Recently, the systematic review of 2 RCTs and 5 observational studies showed that the DTAS strategy was associated with faster onset to treatment times and improved functional outcomes.^12^ We used data from 1 single-center RCT (ANGIOCAT) instead of pooled data because of the heterogeneity of outcome measures and included patients. Second, our model did not consider adverse events in the DTAS group for non-LVO patients because ANGIOCAT results reported no procedural complication in the DTAS group.^6^ This, again, emphasizes that the DTAS strategy should focus on highly suspected LVO instead of all stroke patients to avoid unnecessary invasive angiographic examination. Notably, flat-panel CT angiography could potentially avoid unnecessary invasive angiography. Third, because of the lack of cost parameters, we could not estimate an ICER of introducing the DTAS strategy with prehospital RACE assessment compared with the current practice in the Netherlands, such as the ITER without prehospital RACE. Calculation of costs associated with implementing the RACE scale is complex because the RACE score could also influence patient routing decisions. For example, patients with a high likelihood of having an LVO (RACE >4) could be transported directly to a comprehensive stroke center to avoid the transfer of patients to hospitals without EVT capabilities.^43^ Hence, costs related to the implementation of the RACE scale should be considered as shared costs among different strategies in acute stroke management. In addition, we did not analyze the cost-effectiveness of the DTAS strategy during daytime and nighttime, indicating around-the-clock DTAS availability. However, the ANGIOCAT trial excluded 70 patients because of an unavailable angiography suite and 120 off-hours admission patients because the neurovascular team could not be onsite before patient arrival.^6^ Therefore, only a small number of selected patients could benefit because of the capacity of the angiography suite and personnel. When evidence from the ongoing trials becomes available, the effect of the DTAS strategy during off-hours should be taken into account in economic evaluations. Finally, this study was conducted using the Dutch health care perspective, and only direct medical costs were included in the model. Therefore, the burden of stroke may have been underestimated. However, the average age of patients with suspected LVO was 74 years in our study, and costs related to return to work do not apply in our cohort. Future studies on a younger population may include indirect costs such as productivity loss.

Personnel factors should be considered to maximize the effect of DTAS in clinical practice. The hospital should take into account the capacity of the angiography team to prevent delays for other patients because of occupied interventionalists for non-LVO patients in the DTAS strategy. Furthermore, the angiography team must be well-trained before introducing the DTAS strategy. Sulzenko et al.^44^ reported that the lack of trained personnel during off-hours resulted in a small number of patients who could be treated according to the DTAS strategy. The benefit of time-saving in the DTAS strategy will be lost if there is not enough medical staff in the angiography team or not well-trained staff.

This study demonstrates that the DTAS strategy may be a cost-effective approach in patients with suspected LVO (RACE >4 and NIHSS >10) when compared with the ITER strategy from the Dutch health care perspective. With faster treatment, improved functional outcomes, and favorable economic evaluation, the DTAS, combined with prehospital triage for LVO, should be considered a potential strategy to optimize the acute stroke care pathway.

## Glossary

AdViSHE: Assessment of the Validation Status of Health Economic decision models
CTA: CT angiography
DTAS: direct transfer to the angiography suite
EVT: endovascular thrombectomy
FAST: face arm speech time
ICER: incremental cost-effectiveness ratio
ITER: initial transfer to the emergency room
IVT: IV thrombolysis
LVO: large vessel occlusion
mRS: modified Rankin Scale
NIHSS: NIH Stroke Scale
OR: odds ratio
QALY: quality-adjusted life year
RACE: Rapid Arterial Occlusion Evaluation
